# Ocular Distribution of Cyclosporine Following Topical Administration of OTX-101 in New Zealand White Rabbits

**DOI:** 10.1089/jop.2018.0106

**Published:** 2019-09-05

**Authors:** Sidney L. Weiss, William G. Kramer

**Affiliations:** ^1^ Auven Therapeutics, Delray Beach, Florida.; ^2^ i-novion, Inc., Randolph, New Jersey.; ^3^ Kramer Consulting LLC, North Potomac, Maryland.

**Keywords:** dry eye disease, cyclosporine, pharmacokinetic, distribution

## Abstract

***Purpose:*** Evaluate the ocular distribution, tolerability, and systemic exposure of cyclosporine (CsA) in New Zealand white rabbits following topical administration of OTX-101, a novel, clear aqueous nanomicellar solution developed for the treatment of dry eye disease (DED).

***Methods:*** The study design included single- and repeat-dose phases. In the single-dose phase, rabbits received a single instillation of OTX-101 0.05% or CsA ophthalmic emulsion 0.05% (Restasis^®^; Allergan, Irvine, CA) as a comparator. In the repeat-dosing phase, OTX-101 (0.01%, 0.05%, or 0.1% CsA) or comparator was instilled 4 times per day for 7 days. Samples collected included whole blood, tears, and ocular tissues/fluids (aqueous humor, choroid-retina, conjunctiva, cornea, superior eyelid, third eyelid, iris/ciliary body, lacrimal gland, lens, sclera, and vitreous humor). CsA concentrations were analyzed using liquid chromatography-tandem mass spectrometry.

***Results:*** Analysis included samples from 112 rabbits. The highest concentration of CsA following a single OTX-101 0.05% instillation occurred in the third eyelid (C_max_ = 1,200 ng/g). Concentrations of CsA in the cornea and superior bulbar conjunctiva increased in a dose-related manner following repeated administration of OTX-101 formulations; C_max_ [T_max_ (h)] for cornea was 1,543 ng/g (6.50), 5,410 ng/g (7.0), and 8,123 ng/g (6.50), for 0.01%, 0.05%, and 0.1% CsA concentrations, respectively; for superior bulbar conjunctiva was 726 ng/g (6.50), 1,468 ng/g (6.50), and 2,080 ng/g (6.25), respectively.

***Conclusions:*** OTX-101 topical ophthalmic instillation resulted in extensive distribution of CsA in ocular tissues, particularly in target tissues for DED (cornea and conjunctiva), while systemic exposure was negligible.

## Introduction

Cyclosporine (CsA) is a fungal-derived peptide immunomodulatory agent with immunosuppressant activities. Cyclosporine inhibits calcineurin, preventing the activation of T lymphocytes. Anti-inflammatory effects of CsA are associated with inhibition of cell-mediated release of proinflammatory cytokines.^1,2^ Systemic administration of CsA has been used for organ recipients and in the treatment of autoimmune disorders, due to the immunosuppressant properties of the molecule.^3^ Topical ophthalmic administration of CsA is utilized in the United States for the treatment of dry eye disease (DED), a common ocular condition associated with alterations in the tear film and ocular surface inflammation.^4–6^ Cyclosporine ophthalmic emulsion 0.05% (Restasis^®^; Allergan, Irvine, CA)—which is indicated to increase tear production in patients with DED^7^—significantly increased tear production and reduced the signs and symptoms of DED following topical ocular administration.^8^

Poor aqueous solubility of CsA has led to the development of alternative approaches in the formulation of the agent for delivery to the ocular surface. OTX-101 (Cequa™; Sun Pharmaceutical Industries, Inc., Cranbury, NJ) is a novel, nanomicellar, clear aqueous solution of CsA. OTX-101 was approved in August 2018 by the U.S. Food and Drug Administration (FDA) to increase tear production in patients with keratoconjunctivitis sicca. The safety and efficacy of OTX-101 have been evaluated through late-stage clinical investigations. OTX-101 administration significantly reduced ocular surface staining (both cornea and conjunctiva) relative to vehicle in subjects with DED. In addition, a higher proportion of subjects in the OTX-101 treatment groups demonstrated a ≥10 mm increase in unanesthetized Schirmer Tear Test score over a 12-week treatment period compared to the vehicle group. Ocular administration of OTX-101 was well tolerated.^9^

The objective of this nonclinical pharmacokinetic (PK) study was to evaluate the ocular distribution, tolerability, and systemic exposure of CsA following topical administration of OTX-101.

## Methods

### Experimental design

The study was divided into 2 phases (Table 1). The first phase involved topical ophthalmic administration of a single dose of study drug, followed by terminal collection of tissues and fluids for up to 72 h. The second phase of the study was a repeat-dose phase, in which each animal received multiple topical instillations of study drug before terminal collection of tissues and fluids for up to 18 h after the last dosing on day 7. Both phases of the study were designed to evaluate the ocular distribution, tolerability, and systemic distribution of CsA.

**Table 1.** Randomized Treatment Assignment

*Study group*	*Cyclosporine concentration (%)*	*No. of rabbits*
Untreated control	—	2
Single-dose phase		
OTX-101	0.05	20
Comparator^a^	0.05	20
Repeat-dose phase		
OTX-101 low dose	0.01	10
OTX-101 mid-dose	0.05	20
OTX-101 high dose	0.1	20
Comparator^a^	0.05	20

Each animal was assigned to a group using a randomized block design based on body weight. Animals in the control group did not receive any treatment. Single-dose phase groups received 1 bilateral instillation of study drug. Animals in the repeat-dose phase groups received 4 bilateral instillations at ∼2-h intervals (±5 min) for up to 7 consecutive days.

^a^ Comparator = cyclosporine ophthalmic emulsion 0.05% (Restasis; Allergan, Irvine, CA).

### Animals

A total of 112 New Zealand white female rabbits were used in this study. Animals were housed under standard conditions and allowed an acclimation period of 3–4 weeks between receipt and starting the treatment phases of the study. The general health and ocular condition of each animal were evaluated by a veterinarian before initiation of treatment. The study plan was reviewed and approved by the Animal Care Committee of the laboratory where the study was conducted. All rabbits used in this study were cared for in accordance with the principles outlined in the current “Guide for the Care and Use of Laboratory Animals, Eighth Edition” published by the National Institutes of Health. The authors confirm that use of animals in this study adhered to the Association for Research in Vision and Ophthalmology statement for the Use of Animals in Ophthalmic and Vision Research.

### Study drugs

The test agent, OTX-101—an aqueous solution at concentrations of 0.01%, 0.05%, or 0.1%—was evaluated in this study. These concentrations correspond to an equivalent dose of 3.5, 17.5, and 35 μg of CsA in a 35 μL instillation, respectively. The comparator, CsA ophthalmic emulsion 0.05%, was obtained from commercially available supplies (Restasis; Allergan, Irvine, CA). The CsA ophthalmic emulsion was removed from the commercial container and placed into sterile glass vials to facilitate dispensing using a calibrated pipette. A new pipette tip was used for each administration of both study drugs. The OTX-101 formulations were analyzed by the manufacturer before shipment and a certificate of analysis was provided for each formulation used. Samples of the OTX-101 formulation were analyzed at the end of the study to confirm CsA stability during the study conduct.

### Drug administration

Each animal was assigned to a group using a randomized block design based on body weight. Rabbits were randomly assigned to treatment groups of 10 or 20, in order to provide 2 animals per sampling time point (Table 1). A control group (2 rabbits) did not receive any treatment. In the single-dose phase of the study, the ocular distribution and systemic exposure of CsA were evaluated following a single bilateral instillation of either OTX-101 (0.05%) or CsA ophthalmic emulsion (0.05%) as a comparator. In the repeat-dose phase of the study, rabbits received 4 bilateral instillations to the ocular surface per day for up to 7 consecutive days of either OTX-101 0.01%, 0.05%, or 0.1%, or CsA ophthalmic emulsion 0.05%, administered at 2-h intervals (±5 min). A calibrated pipette was used to deliver 35 μL of study drug (OTX-101 or CsA ophthalmic emulsion) to the ocular surface (cornea and conjunctiva) of each eye. Sampling times for the single-dose and repeat-dose phases of the study are presented in Table 2.

**Table 2.** Sample Collection Time Points for Pharmacokinetic Analysis

*Study group*	*Sample collection time*
Untreated control	Predose
Single-dose phase	
OTX-101 0.05%	Postdose: 0.25, 0.5, 1, 2, 4, 8, 12, 24, 48, and 72 h
Comparator^a^	Postdose: 0.25, 0.5, 1, 2, 4, 8, 12, 24, 48, and 72 h
Repeat-dose phase	
OTX-101 0.01%	Day 7: 2 h after the third dose; 0.25, 0.5, 1, and 2 h after the fourth dose
OTX-101 0.05%	Day 4: predose (0 h)
	Day 7: predose (0 h); 2 h after the third dose; 0.25, 0.5, 1, 2, 4, 8, and 18 h after the fourth dose
OTX-101 0.1%	Day 4: predose (0 h)
	Day 7: predose (0 h); 2 h after the third dose; 0.25, 0.5, 1, 2, 4, 8, and 18 h after the fourth dose
Comparator^a^	Day 4: predose (0 h)
	Day 7: predose (0 h); 2 h after the third dose; 0.25, 0.5, 1, 2, 4, 8, and 18 h after the fourth dose

Animals in the control group did not receive any treatment and served as the predose samples. Rabbits in the single-dose phase groups received 1 bilateral instillation of study drug. Animals in the repeat-dose phase groups received 4 bilateral instillations at ∼2-h intervals (±5 min) for up to 7 consecutive days. Samples were collected from 2 animals in each treatment group at each time point.

^a^ Comparator = cyclosporine ophthalmic emulsion 0.05% (Restasis; Allergan, Irvine, CA).

### Tolerability assessments

Cage-side clinical observations were conducted once daily during the animal acclimation and treatment period for rabbits assigned to the single-dose groups. Clinical signs were assessed twice daily for animals in the repeat-dosing treatment groups, once before the first daily dose and 30 min to 1 h after the last administration of study drug for the day. Detailed clinical evaluations were performed pretreatment, once weekly during the acclimation period and before euthanasia.

### Sample collection

At the scheduled sampling time, 2 animals were randomly selected from each treatment group for collection of ocular tissues, ocular fluids, and whole blood. A Schirmer strip (Alcon Laboratories, Fort Worth, TX) was used to collect tear samples. The Schirmer tear test strip was placed in the lower conjunctival sac and the eyelids held closed until the strip were saturated. Tear samples were placed in a separate labeled vial on dry ice until storage at ≤ −60°C before analysis. Whole blood samples (∼6 mL) were collected by cardiac puncture under anesthesia just before euthanasia. Blood samples were divided into 2 aliquots, placed on dry ice, and moved to storage at ≤ −60°C before CsA analysis. Rabbits were euthanized by an intravenous dose of sodium pentobarbital. Immediately after euthanasia, superior bulbar conjunctiva, third eyelid, and superior eyelid were collected from both eyes of each animal, followed by enucleation and removal of the lacrimal gland. Samples were frozen immediately by flash freezing in liquid nitrogen and then stored at ≤ −60°C until dissection; this prevented postmortem migration of the drug between tissues. Frozen eyes were dissected to isolate the ocular tissues and ocular fluids, including aqueous humor, vitreous humor, cornea, lens, iris/ciliary body, choroid/retina, and sclera. Samples were placed in preweighed vials, then reweighed, and stored at ≤ −60°C until analysis.

### Sample analysis for CsA

Samples were shipped on dry ice to a bioanalytical laboratory (Intertek Pharmaceutical Services, El Dorado Hills, CA) for CsA quantification. Each tissue was analyzed using a validated liquid chromatography-tandem mass spectrometry (LC-MS/MS) method. The lower limit of quantitation (LLOQ) for CsA in whole blood was 0.05 ng/mL; 0.5 ng/mL in aqueous humor and vitreous humor; 0.01 ng in choroid/retina, lens, and lacrimal gland; 0.05 ng in cornea, iris/ciliary body, sclera, conjunctiva, and eyelids; and 0.5 ng in tears. The analyses were performed separately for each sample matrix. Cyclosporine was extracted from Schirmer tear test strips by incubating the strips in 1.25 mL solvent (50:50 acetonitrile: 5 mM ammonium acetate) and diluting the supernatant for analysis. The amount of CsA in the test strips was normalized to sample weights to determine the concentration results. Concentrations of CsA and an internal standard (cyclosporine-d_4_) in whole blood samples were determined by liquid-liquid extraction and subsequent analysis by LC-MS/MS. Concentrations of CsA in aqueous humor and vitreous humor were evaluated by protein precipitation with methanol followed by analysis of the supernatant by LC-MS/MS. The amount of CsA in ocular tissue samples was determined by bead homogenization in extraction buffer (20:80 acetonitrile: 50 mM ammonium acetate, pH 3.5) and protein precipitation extraction using acetonitrile with 0.1% formic acid. An aliquot of the supernatant was extracted further using a phospholipid removal plate and analyzed by a validated LC-MS/MS method.

### PK parameters

Cyclosporine concentrations in whole blood and ocular tissues and fluids at each nominal sampling time were averaged by dose and sample collection time point to create composite animals for PK analysis. Due to the use of composite animals, nominal sampling times were used in all PK analyses. Samples that were identified as <LLOQ were taken as 0. Ocular tissue samples were obtained from both eyes for each rabbit; therefore, each animal contributed 2 samples to the composite concentrations.

The analysis of the PK parameters was conducted using noncompartmental methods. Maximum CsA concentrations (C_max_) and time to the maximum concentration (T_max_) in the whole blood or ocular samples were obtained directly from the composite data. Area under the curve from 0 to the last sample with a composite concentration ≥LLOQ [AUC_(0−t)_] was calculated using the linear trapezoidal method, which is the standard methodology accepted by the FDA. Statistical analysis and PK interpretation were performed using SAS^®^ for Windows, version 9.1.3 (Cary, NC).

## Results

A total of 2,799 samples consisting of whole blood (*n* = 112) and ocular tissues or fluids (*n* = 2,687) were received for analysis at the bioanalytical laboratory. All whole blood samples were included in the analysis. Twenty-four samples (0.9%) of ocular tissue/fluids were excluded from the analysis due to errors in sample processing, labeling discrepancies, or samples not being received. All time points had at least 2 replicate samples for the analysis, allowing a sufficient number of samples to calculate the composite animal PK parameters.

### PK analysis

#### Single-dose phase

**FIG. 1.**
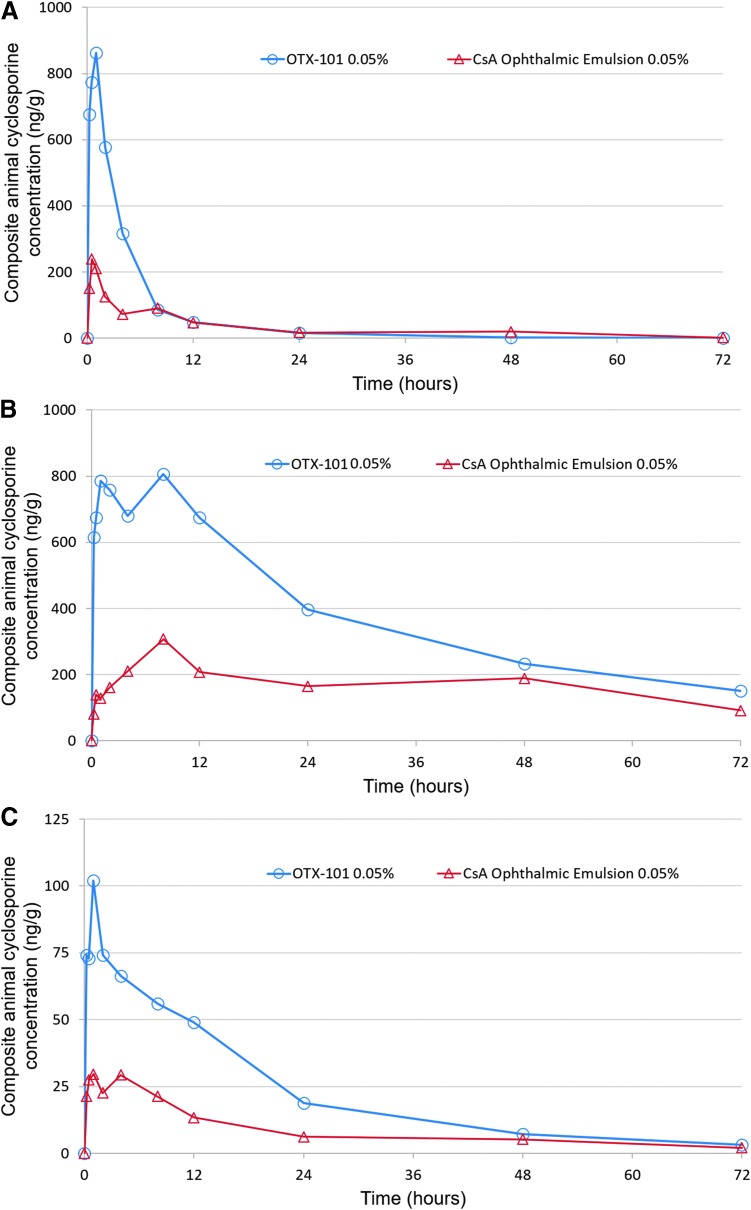
Composite animal cyclosporine concentrations in superior bulbar conjunctiva **(A)**, cornea **(B)**, and sclera **(C)** after a single topical ocular instillation of OTX-101 0.05% (*open circles*) or cyclosporine ophthalmic emulsion 0.05% (*open triangles*) to New Zealand white rabbits. Cyclosporine ophthalmic emulsion 0.05% (Restasis; Allergan, Irvine, CA) was used as a comparator. Samples were collected at the following time points after instillation: 0.25, 0.5, 1, 2, 4, 8, 12, 24, 48, and 72 h. CsA, cyclosporine.

Cyclosporine concentrations in all whole blood and ocular tissue/fluid samples were below the LLOQ for the untreated control animals. A single bilateral topical instillation of either OTX-101 0.05% or the comparator, CsA ophthalmic emulsion 0.05%, resulted in extensive distribution of CsA into ocular tissues. Minimal systemic exposure to CsA was detected in the whole blood samples. The concentrations of CsA in the composite animal superior bulbar conjunctiva, cornea, and sclera after a single instillation of either OTX-101 0.05% or CsA ophthalmic emulsion 0.05% are presented in Fig. 1. The PK parameters for the distribution of CsA in ocular tissues/fluids and whole blood after single instillation are summarized in Table 3.

**Table 3.** Composite Animal Pharmacokinetic Parameters for Cyclosporine After a Single Topical Ocular Administration of OTX-101 0.05% or Cyclosporine Ophthalmic Emulsion 0.05% to New Zealand White Rabbits

*Matrix*	*Parameter*	*OTX-101 0.05%*	*Comparator*^a^	*Ratio*^b^
Cornea	C_max_ (ng/g)	807	307	
	T_max_ (h)	8.00	8.00	
	AUC_(0−t)_ (h × ng/g)	27,324	12,532	2.18
Superior bulbar conjunctiva	C_max_ (ng/g)	862	239	
	T_max_ (h)	1.00	0.50	
	AUC_(0−t)_ (h × ng/g)	3,890	2,210	1.76
Tears	C_max_ (ng/g)	1,637	3,409	
	T_max_ (h)	0.25	0.50	
	AUC_(0−t)_ (h × ng/g)	7,273	28,323	0.26
Sclera	C_max_ (ng/g)	102	30	
	T_max_ (h)	1.00	1.00	
	AUC_(0−t)_ (h × ng/g)	1,593	613	2.60
Lacrimal gland	C_max_ (ng/g)	22	17	
	T_max_ (h)	0.25	0.50	
	AUC_(0−t)_ (h × ng/g)	322	261	1.23
Aqueous humor	C_max_ (ng/mL)	3	7	
	T_max_ (h)	48.00	24.00	
	AUC_(0−t)_ (h × ng/mL)	288	132	2.17
Vitreous humor	C_max_ (ng/mL)	0	0.71	
	T_max_ (h)	0	0.25	
	AUC_(0−t)_ (h × ng/mL)	0	0.43	—
Superior eyelid	C_max_ (ng/g)	569	1,067	
	T_max_ (h)	1.00	4.00	
	AUC_(0−t)_ (h × ng/g)	8,300	28,034	0.30
Third eyelid	C_max_ (ng/g)	1,200	285	
	T_max_ (h)	0.25	1.00	
	AUC_(0−t)_ (h × ng/g)	5,935	2,917	2.03
Iris/ciliary body	C_max_ (ng/g)	57	10	
	T_max_ (h)	0.25	1.00	
	AUC_(0−t)_ (h × ng/g)	525	257	2.04
Lens	C_max_ (ng/g)	2	1	
	T_max_ (h)	24.00	48.00	
	AUC_(0−t)_ (h × ng/g)	84	44	1.92
Choroid/retina	C_max_ (ng/g)	31	19	
	T_max_ (h)	0.25	1.00	
	AUC_(0−t)_ (h × ng/g)	327	228	1.44
Whole blood	C_max_ (ng/mL)	1	0.09	
	T_max_ (h)	1.00	2.00	
	AUC_(0−t)_ (h × ng/mL)	3	0.38	9.16

Samples that were identified as below the lower limit of quantitation were imputed as 0. The lower limit of quantitation for CsA in whole blood was 0.05 ng/mL; 0.5 ng/mL in aqueous humor and vitreous humor; 0.01 ng in choroid/retina, lens, and lacrimal gland; 0.05 ng in cornea, iris/ciliary body, sclera, conjunctiva, and eyelids; and 0.5 ng in tears.

^a^ Cyclosporine ophthalmic emulsion 0.05% (Restasis; Allergan, Irvine, CA) was used as the comparator.

^b^ Ratio of AUC_(0−t)_ for OTX-101 0.05% to cyclosporine ophthalmic emulsion 0.05%.

AUC_(0−t)_, area under the concentration-time curve from 0 to the last sample; C_max_, maximum cyclosporine concentration; CsA, cyclosporine; T_max_, time to the maximum cyclosporine concentration.

The rank order of exposure as measured by AUC_(0−t)_ in ocular tissues and ocular fluids after a single instillation of OTX-101 0.05% was as follows: cornea > superior eyelid > tears > third eyelid > superior bulbar conjunctiva > sclera > iris/ciliary body > choroid/retina and lacrimal gland > aqueous humor > lens > whole blood (Table 3). All concentrations of CsA in the vitreous humor were below LLOQ following a single administration of OTX-101 0.05%. Concentrations of CsA were higher in all ocular tissues and fluids after a single administration of OTX-101 0.05% compared to CsA ophthalmic emulsion 0.05%, except for the superior eyelid and tears, as illustrated by the AUC_(0−t)_ ratios presented in Table 3.

#### Repeat-dose phase

A similar profile in the ocular distribution of CsA was observed in the samples from the repeat-dosing phase of the study. Cyclosporine was extensively distributed in ocular tissues and fluids following repeated dosing (4 times per day at 2-h intervals) of either OTX-101 (0.01%, 0.05%, or 0.1%) or the comparator, CsA ophthalmic emulsion 0.05%. Systemic exposure to CsA increased above the level detected in the single-dose phase, but remained minimal. PK parameters for the distribution of CsA in ocular tissues/fluids and whole blood following repeat dosing of OTX-101 (0.01%, 0.05%, and 0.1%) or CsA ophthalmic emulsion 0.05% are summarized in Table 4.

**Table 4.** Composite Animal Pharmacokinetic Parameters for Cyclosporine After Repeated Topical Ocular Administration (4 Applications Per Day for 7 Days) of OTX-101 (0.01%, 0.05%, or 0.1%) or the Comparator, Cyclosporine Ophthalmic Emulsion 0.05%, to New Zealand White Rabbits

*Matrix*	*Parameter*	*OTX-101 0.01%*	*OTX-101 0.05%*	*OTX-101 0.1%*	*Comparator*^a^
Cornea	C_max_ (ng/g)	1,543	5,410	8,123	3,075
	T_max_ (h)	6.50	7.00	6.50	6.25
	AUC_(0−t)_ (h × ng/g)	2,837	72,003	105,640	36,011
Superior bulbar conjunctiva	C_max_ (ng/g)	726	1,468	2,080	1,462
	T_max_ (h)	6.50	6.50	6.25	6.25
	AUC_(0−t)_ (h × ng/g)	987	6,778	9,679	5,890
Tears	C_max_ (ng/g)	3,429	2,452	4,173	9,420
	T_max_ (h)	8.00	6.25	6.50	6.00
	AUC_(0−t)_ (h × ng/g)	3,518	15,649	28,552	28,588
Sclera	C_max_ (ng/g)	94	261	592	166
	T_max_ (h)	6.25	6.50	7.00	6.25
	AUC_(0−t)_ (h × ng/g)	164	2,579	5,010	1,398
Lacrimal gland	C_max_ (ng/g)	78	64	271	141
	T_max_ (h)	6.50	7.00	8.00	6.50
	AUC_(0−t)_ (h × ng/g)	92	450	1,483	1,002
Aqueous humor	C_max_ (ng/mL)	13	52	114	28
	T_max_ (h)	6.50	6.50	10.00	6.50
	AUC_(0−t)_ (h × ng/mL)	22	787	1,407	334
Vitreous humor	C_max_ (ng/mL)	0.14	1	3	0.72
	T_max_ (h)	6.25	7.00	6.50	6.50
	AUC_(0−t)_ (h × ng/mL)	0.02	16	26	3
Superior eyelid	C_max_ (ng/g)	2,450	6,458	10,518	12,795
	T_max_ (h)	6.50	6.50	24.00	7.00
	AUC_(0−t)_ (h × ng/g)	2,602	41,092	106,250	118,504
Third eyelid	C_max_ (ng/g)	727	2,003	3,460	934
	T_max_ (h)	6.25	6.25	6.25	6.25
	AUC_(0−t)_ (h × ng/g)	916	8,906	17,118	5,807
Iris/ciliary body	C_max_ (ng/g)	32	138	391	69
	T_max_ (h)	6.50	14.00	10.00	8.00
	AUC_(0−t)_ (h × ng/g)	51	2,070	3,799	721
Lens	C_max_ (ng/g)	11	61	126	22
	T_max_ (h)	8.00	24.00	14.00	6.25
	AUC_(0−t)_ (h × ng/g)	21	985	2,169	324
Choroid/retina	C_max_ (ng/g)	22	67	158	54
	T_max_ (h)	7.00	7.00	7.00	7.00
	AUC_(0−t)_ (h × ng/g)	39	855	1,536	431
Whole blood	C_max_ (ng/mL)	0.33	2	6	0.56
	T_max_ (h)	6.25	6.50	6.50	6.00
	AUC_(0−t)_ (h × ng/mL)	0.48	7	16	3

^a^ Cyclosporine ophthalmic emulsion 0.05% (Restasis; Allergan, Irvine, CA) was used as the comparator.

**FIG. 2.**
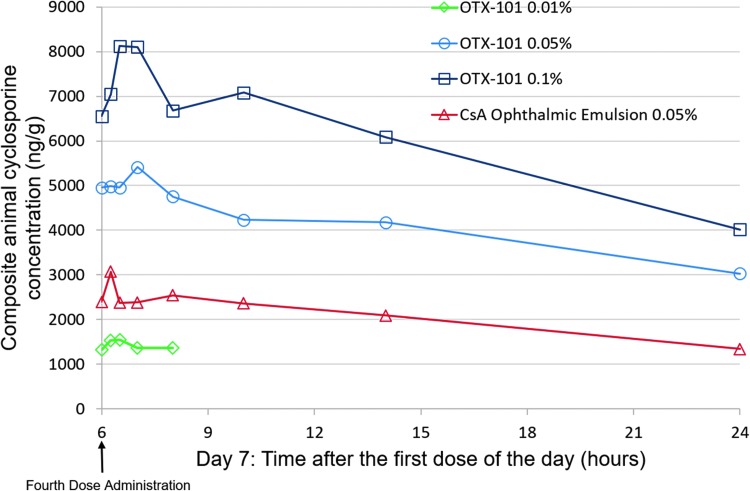
Composite animal cyclosporine concentrations in the cornea following repeat dosing for 7 consecutive days of OTX-101 0.01% (*open diamonds*), OTX-101 0.05% (*open circles*), OTX-101 0.1% (*open squares*), or cyclosporine ophthalmic emulsion 0.05% (*open triangles*) to New Zealand white rabbits. Cyclosporine ophthalmic emulsion 0.05% (Restasis; Allergan, Irvine, CA) was used as a comparator. Samples were collected at the following time points after the fourth (hour 6) dose of OTX-101 0.05%, OTX-101 0.1%, and the comparator on day 7: 0.25, 0.5, 1, 2, 4, 8, and 18 h. Samples were collected at the following time points after the fourth dose of OTX-101 0.01% on day 7: 0.25, 0.5, 1, and 2 h.

Cyclosporine concentrations in ocular tissues/fluids and whole blood samples collected before administration of the first dose on day 4 were comparable to the CsA concentrations in samples collected before the first dose on day 7. Concentration of CsA in ocular tissues and fluids increased in a generally dose-related manner following instillation of OTX-101 0.01%, 0.05%, and 0.1% (Fig. 2). The AUC_(0−t)_ ratio comparisons between samples from the OTX-101 0.05%- and OTX-101 0.1%-administered animals ranged from 1.43 (superior bulbar conjunctiva) to 3.29 (lacrimal gland), with most tissue comparisons close to a 2-fold increase in dose (Table 4). Ocular tissue, ocular fluid, and whole blood concentrations of CsA were higher after repeated topical administration of OTX-101 0.05% than the comparator, CsA ophthalmic emulsion 0.05%, except for lacrimal gland, superior eyelid, and tears. Concentrations of CsA were higher after repeated topical administration of OTX-101 0.1% versus the comparator in all ocular tissues and fluids, except for the superior eyelid (Table 4). The rank order of exposure based on AUC_(0−t)_ in ocular tissues and ocular fluids after repeated administration of OTX-101 0.05% and OTX-101 0.1% was superior eyelid and cornea > tears > third eyelid > superior bulbar conjunctiva > sclera > iris/ciliary body > lens > choroid/retina, lacrimal gland, and aqueous humor > vitreous humor > whole blood (Table 4). An increase in CsA exposure, as measured by AUC_(0−t)_, was observed in all ocular tissues following repeated administration. The ratios of the repeat-dose AUC_(0−t)_ to single-dose AUC_(0−t)_ ranged from 1.40 for the lacrimal gland to 11.69 for the lens, indicating accumulation in ocular tissues with repeat dosing (Table 5).

**Table 5.** Ratio of Area Under the Concentration-Time Curve from Time 0 to the Last Sample for OTX-101 0.05% Administered as Repeated Versus Single Dose to New Zealand White Rabbits

*Matrix*^a^	*Single-dose*	*Repeat-dose*	*Ratio (repeat/single)*
	*OTX-101 0.05%*	*OTX-101 0.05%*	
Cornea	27,324	72,003	2.64
Superior bulbar conjunctiva	3,891	6,778	1.74
Tears	7,273	15,649	2.15
Sclera	1,593	2,579	1.62
Lacrimal gland	322	450	1.40
Aqueous humor	288	787	2.73
Vitreous humor	0	16	—
Superior eyelid	8,300	41,092	4.95
Third eyelid	5,935	8,906	1.50
Iris/ciliary body	525	2,070	3.94
Lens	84	985	11.68
Choroid/retina	327	855	2.61
Whole blood	3	8	2.19

Rabbits in the single-dose phase of the study received 1 bilateral instillation of study drug. The repeat-dose phase animals received 4 bilateral instillations at ∼2-h intervals (±5 min) for up to 7 consecutive days. Samples that were identified as below the lower limit of quantitation were imputed as 0. The lower limit of quantitation for CsA in whole blood was 0.05 ng/mL; 0.5 ng/mL in aqueous humor and vitreous humor; 0.01 ng in choroid/retina, lens, and lacrimal gland; 0.05 ng in cornea, iris/ciliary body, sclera, conjunctiva, and eyelids; and 0.5 ng in tears.

^a^ Units for all ocular tissue AUC_(0−t)_ are h × ng/g and h × ng/mL for aqueous humor, vitreous humor, and whole blood.

AUC_(0-t)_, area under the concentration-time curve from 0 to the last sample; CsA, cyclosporine.

### Safety assessments

#### Tolerability

No treatment-related clinical signs were observed following administration of either OTX-101 or the comparator, CsA ophthalmic emulsion 0.05%. No mortality occurred during the conduct of the study. Body weights were unaffected by treatment and were comparable to control animals.

## Discussion

The product, OTX-101, evaluated in this study is intended for topical ocular treatment of DED. From pioneering work by Stern et al. describing the pathogenesis of immune-mediated DED, it is known that for the treatment of DED, the target tissue sites of action for CsA are the conjunctiva and cornea.^10,11^ Understanding PK at the site of action, demonstrating free drug exposure in these tissues, and determining if the drug achieves desired target concentrations are important factors in drug development.^12^ For CsA to inhibit calcineurin in target tissues, an estimated tissue concentration of 10–20 μg/g has to be achieved.^13^ However, for topical ocular drugs administered by the patient, suboptimal therapy is expected due to anticipated poor patient treatment adherence; therefore, in topical ocular drug development, the highest practical target tissue concentrations that can be achieved without causing acute or chronic toxicity are desired.^14^ In this nonclinical PK study, OTX-101 resulted in approximately double the CsA concentration in the conjunctiva and cornea after a single (0.05%) or repeat (0.05% and 0.1%) dosing compared with the commercially available CsA ophthalmic emulsion 0.05%. There were comparable levels of CsA in other DED-relevant tissues or fluids, such as tear film, aqueous humor, and lacrimal gland, following single and repeat doses of OTX-101 and CsA ophthalmic emulsion 0.05%, with very low concentrations in the peripheral blood (C_max_ <7 ng/mL). These higher concentrations of ocular surface CsA after OTX-101 dosing may translate to a better clinical response after treatment with OTX-101 in DED compared with CsA ophthalmic emulsion 0.05%; however, additional studies are needed to confirm this hypothesis.

Despite rapid and extensive distribution of CsA in ocular tissues of the anterior segment following topical ocular administration of OTX-101 in this study, there were no signs of toxicity. The dosing was well tolerated, despite the higher concentrations of CsA attained in most ocular tissues after OTX-101 compared to CsA ophthalmic emulsion 0.05%. Ocular tissues and ocular fluid concentrations of CsA were observed to increase in a generally dose-related manner following instillation of OTX-101 0.01%, 0.05%, and 0.1%, suggesting that the novel aqueous formulation results in increased target tissue concentrations of CsA compared with the commercially available CsA suspension. In addition, a ratio of the repeat-dose (4 times a day for 7 days) AUC_(0−t)_ to single-dose AUC_(0−t)_ ranged from 1.40 for the lacrimal gland to 11.68 for the lens for OTX-101, and 2.28 for sclera and 7.35 for the lens for the comparator, CsA ophthalmic emulsion 0.05% (data not shown), indicating accumulation in ocular tissues with repeat dosing with both formulations. Accumulation of the drug in tissue occurs if drug intake exceeds drug elimination, especially when a drug is repeatedly administered.^15^ Although toxicity was not observed with these CsA concentrations, the observed drug accumulation suggests that 4 times a day dosing may be excessive, may lead to accumulation, and may increase the chance of development of toxicity.

A limitation of this study is that the *in vivo* methodology used is not always predictive of the tissue penetration in a clinical setting. However, the corresponding clinical PK studies that would address this limitation cannot ethically be carried out as they would involve potential damage to the subject's eyes.

In conclusion, the results of this study indicate that OTX-101 was well tolerated following single and repeated administrations and produced higher concentrations of CsA in the cornea, conjunctiva, and most other ocular tissues, than the comparator, the currently marketed CsA ophthalmic emulsion 0.05%. Systemic exposure was minimal following topical ophthalmic administration of OTX-101.

## References

[1] Pflugfelder S.C. Antiinflammatory therapy for dry eye. Am. J. Ophthalmol. 137:337–342, 2004 14962426 10.1016/j.ajo.2003.10.036

[2] Fruman D.A., Klee C.B., Bierer B.E., and Burakoff S.J. Calcineurin phosphatase activity in T lymphocytes is inhibited by FK 506 and cyclosporin A. Proc. Natl. Acad. Sci. U.S.A. 89:3686–3690, 1992 1373887 10.1073/pnas.89.9.3686 PMC525555

[3] Moore C.P., McHugh J.B., Thorne J.G., and Phillips T.E. Effect of cyclosporine on conjunctival mucin in a canine keratoconjunctivitis sicca model. Invest. Ophthalmol. Vis. Sci. 42:653–659, 2001 11222523

[4] Bron A.J., De Paiva C.S., Chauhan S.K., et al. TFOS DEWS II Pathophysiology Report. Ocul. Surf. 15:438–510, 2017 28736340 10.1016/j.jtos.2017.05.011

[5] Stern M.E., Schaumburg C.S., and Pflugfelder S.C. Dry eye as a mucosal autoimmune disease. Int. Rev. Immunol. 32:19–41, 2013 23360156 10.3109/08830185.2012.748052 PMC3587314

[6] Subcommittee of the International Dry Eye Workshop. The definition and classification of dry eye disease: report of the Definition and Classification Subcommittee of the International Dry Eye Workshop (2007). Ocul. Surf. 5:75–92, 2007 17508116 10.1016/s1542-0124(12)70081-2

[7] Restasis^®^ (cyclosporine ophthalmic emulsion) 0.05%, for topical ophthalmic use. Full prescribing information. Irvine, CA: Allergan, 2017

[8] Sall K., Stevenson O.D., Mundorf T.K., Reis B.L., and the CsA Phase 3 Study Group. Two multicenter, randomized studies of the efficacy and safety of cyclosporine ophthalmic emulsion in moderate to severe dry eye disease. Ophthalmology. 107:631–639, 2000 10768324 10.1016/s0161-6420(99)00176-1

[9] Tauber J., Schechter B.A., Bacharach J., et al. A phase 2/3, randomized, double-masked, vehicle-controlled, dose-ranging study of the safety and efficacy of OTX-101 in the treatment of dry eye disease. Clin. Ophthalmol. 12:1921–1929, 2018 30323548 10.2147/OPTH.S175065 PMC6174300

[10] Stern M.E., and Pflugfelder S.C. Pathogenesis: emphasis on dry eye and the lacrimal functional unit in Sjogren's Syndrome. In: Fox R., and Fox C., eds. Sjogren's Syndrome. New York: Springer; 2011; p. 203–220

[11] Stern M.E., Schaumburg C.S., Dana R., et al. Autoimmunity at the ocular surface: pathogenesis and regulation. Mucosal Immunol. 3:425–442, 2010 20485329 10.1038/mi.2010.26 PMC3577924

[12] Rizk M.L., Zou L., Savic R.M., and Dooley K.E. Importance of drug pharmacokinetics at the site of action. Clin. Transl. Sci. 10:133–142, 2017 28160433 10.1111/cts.12448 PMC5421734

[13] Halloran P.F., Helms L.M., Kung L., and Noujaim J. The temporal profile of calcineurin inhibition by cyclosporin in vivo. Transplantation. 68:1356–1361, 1999 10573076 10.1097/00007890-199911150-00023

[14] Gooch N., Molokhia S.A., Condie R., et al. Ocular drug delivery for glaucoma management. Pharmaceutics. 4:197–211, 2012 24300188 10.3390/pharmaceutics4010197 PMC3834906

[15] van Rossum J.M. Pharmacokinetics of accumulation. J. Pharm. Sci. 57:2162–2165, 1968 5708363 10.1002/jps.2600571230

